# World Heart Federation Roadmap for Digital Health in Cardiology

**DOI:** 10.5334/gh.1141

**Published:** 2022-08-26

**Authors:** Jasper Tromp, Devraj Jindal, Julie Redfern, Ami Bhatt, Tania Séverin, Amitava Banerjee, Junbo Ge, Dipti Itchhaporia, Tiny Jaarsma, Fernando Lanas, Francisco Lopez-Jimenez, Awad Mohamed, Pablo Perel, Gonzalo Emanuel Perez, Fausto Pinto, Rajesh Vedanthan, Axel Verstrael, Khung Keong Yeo, Kim Zulfiya, Dorairaj Prabhakaran, Carolyn S. P. Lam, Martin R. Cowie

**Affiliations:** 1 Saw Swee Hock School of Public Health, National University of Singapore, The National University Health System Singapore, and Duke-NUS Medical school, Singapore; 2 Centre for Chronic Disease Control, New Delhi, IN; 3 School of Health Sciences, Faculty of Medicine and Health, University of Sydney, AU; 4 Massachusetts General Hospital, Boston, US; 5 World Heart Federation, Geneva, CH; 6 Institute of Health Informatics, University College London, London, GB; 7 Fudan University Zhongshan Hospital, CN; 8 Hoag Hospital, University of California, Irvine, Newport Beach, US; 9 Dpt of Medicine, Health and Caring Sciences, Linköping University, Linköping, SE; 10 Universidad de La Frontera, Temuco, CL; 11 Dpt of cardiovascular medicine, Mayo Clinic, Rochester, US; 12 Dpt of Medicine, University of Khartoum, Khartoum, SD; 13 London School of Hygiene & Tropical Medicine, London, UK; 14 Dpt of Cardiology, Clínica Olivos, Buenos Aires, AR; 15 University Hospital CHULN, CAML, CCUL@RISE, Faculty of Medicine of the University of Lisbon, Lisbon, PT; 16 Department of Population Health/Institute for Excellence in Health Equity, New York University Grossman School of Medicine, New York, US; 17 Hasselt University, Antwerp, BE; 18 Dpt of cardiology, National Heart Centre Singapore, SG; 19 Dpt of internal medicine, City Clinical Hospital No. 7, Kazan medical state university, Kazan, RU; 20 Public Health Foundation of India, Gurugram, IN; 21 National Heart Centre Singapore, Duke-National University of Singapore, SG; 22 Royal Brompton Hospital & School of Cardiovascular Medicine, Faculty of Life-sciences and Medicine, King’s College London, London, GB

**Keywords:** digital health interventions for CVD, e-health, health system governance

## Abstract

More than 500 million people worldwide live with cardiovascular disease (CVD). Health systems today face fundamental challenges in delivering optimal care due to ageing populations, healthcare workforce constraints, financing, availability and affordability of CVD medicine, and service delivery.

Digital health technologies can help address these challenges. They may be a tool to reach Sustainable Development Goal 3.4 and reduce premature mortality from non-communicable diseases (NCDs) by a third by 2030. Yet, a range of fundamental barriers prevents implementation and access to such technologies. Health system governance, health provider, patient and technological factors can prevent or distort their implementation.

World Heart Federation (WHF) roadmaps aim to identify essential roadblocks on the pathway to effective prevention, detection, and treatment of CVD. Further, they aim to provide actionable solutions and implementation frameworks for local adaptation. This WHF Roadmap for digital health in cardiology identifies barriers to implementing digital health technologies for CVD and provides recommendations for overcoming them.

## Background

Cardiovascular disease (CVD) affects more than 500 million people worldwide if you wording needs to be modified [1]. Global lifestyle changes, including worsening diets, reduced physical activity and an increasing smoking prevalence, have led to a growing burden of non-communicable diseases (NCDs) and risk factors for CVD [2,3]. Health systems today face fundamental challenges in delivering optimal care due to ageing populations, constraints in their healthcare workforce, financing, availability and affordability of CVD medicine, and service delivery [4,5,6]. Therefore, patient self-care and empowerment is becoming increasingly important.

**Figure 1:**
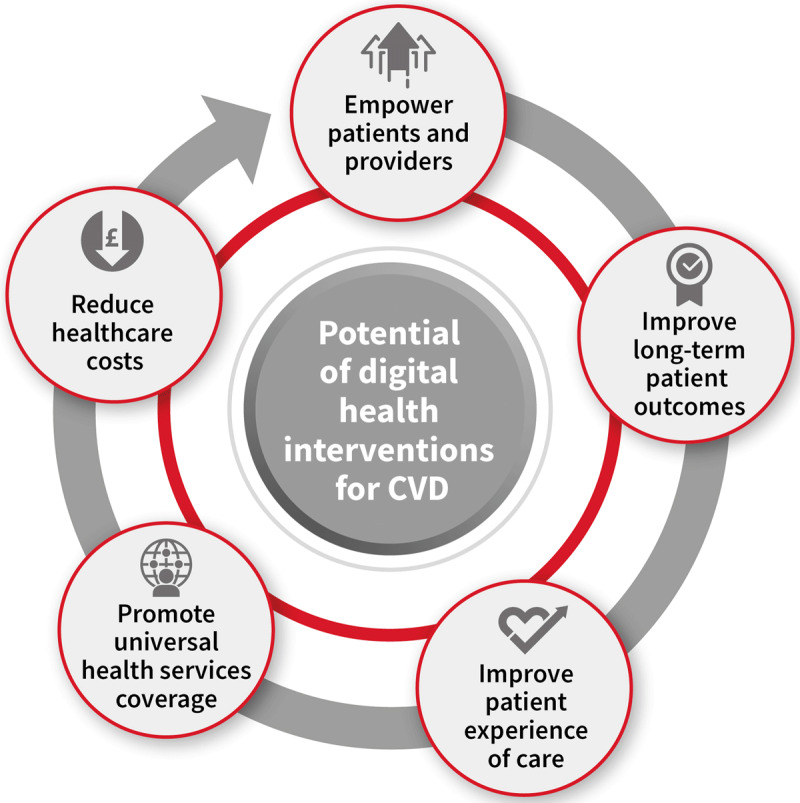
The potential of digital health interventions for CVD. *© World Heart Federation*.

Digital health technologies, such as electronic decision support tools, telemonitoring, remote monitoring, or mobile health (mHealth) apps, have the potential to help address health system challenges which limit the achievement of optimal and universal health coverage (UHC). These technologies can contribute to UHC by empowering patients [7] and providers [8], promoting universal health services coverage [9], improving long-term patient outcomes and care experience, and reducing healthcare costs (Figure 1) [10]. Therefore, Digital Health Interventions (DHIs) may be a tool to reach Sustainable Development Goal 3.4 and reduce premature mortality from NCDs by a third by 2030 [11].

A range of fundamental barriers prevent implementation and access to digital health technologies globally [12]. Health system governance (e.g., national privacy regulations and internet access), health provider (e.g., digital literacy, perceived effectiveness), patient (e.g., age [13], local sex/gender norms [14], socioeconomic factors [15], digital and health literacy [16]), and technological factors (e.g., a context-specific adaptation of technology, interoperability [16]) can prevent or distort the implementation of new digital health technologies. Digital determinants of health and socioeconomic factors determining access and adoption of digital technologies can create a ‘digital divide’: a chasm between those with and without access to digital technologies due to economic factors, (digital and health) literacy, age, or sex. This can cause inequity and inequality in access to digital technology and its potential benefits. Unfortunately, similar inequalities often permeate across diseases and beyond health [17]. Furthermore, it is essential to acknowledge that not all DHIs to date have positively impacted outcomes [18,19]. Process evaluations have demonstrated that common barriers, such as (poor) context-specific design of interventions or poor implementation, are often at the root of an intervention’s effectiveness [20,21]. Yet proper process evaluations for complex DHI are not commonly performed, leaving many questions at the end of effectiveness studies about why DHI were (not) effective. The World Heart Federation (WHF) Roadmaps have been published since 2014 to identify essential roadblocks on the pathway to effective prevention, detection, and treatment of cardiovascular disease [22,23,24,25,26,27,28]. The ultimate aim of the WHF Roadmap is to provide implementation frameworks for local adaptation for prevention, detection and treatment strategies for CVD. This WHF roadmap for digital health in cardiology aims to identify barriers to implementing digital health technologies for CVD and provide recommendations for overcoming them.

## Methodology and selection of the expert writing group

In 2020, the WHF commissioned a writing group to develop a roadmap on digital health for cardiology. WHF regional Members were invited to nominate an expert to the writing group to ensure that the content of the roadmap has accurate global representation. In addition, experts in digital health with clinical, public health or research backgrounds were selected. The writing group also included representatives from allied health professions and people living with CVD to ensure an inclusive perspective.

The roadmap is derived from a synthesis of peer-reviewed evidence on the barriers to and benefits of using digital technologies, an online survey among WHF members and the public and an iterative process of expert consultation involving eight writing committee members and 12 reviewing committee members drawn from the global WHF membership network. The Supplementary Appendix details the methodology of the survey. Inputs from surveys and (patient) experts were supplemented with a narrative evidence synthesis on barriers and solutions for implementing digital health solutions. The Writing Committee identified and selected case studies based on their experience and relevance to the identified barriers and solutions.

## Target audience

The primary focus of this roadmap is to identify and provide solutions and tools for commonly faced barriers in the development, implementation and scaling of DHIs for CVDs and their risk factors. Solutions in this roadmap are intended to be practical and are directed at different stakeholders within health systems, including people living with CVD and who might be active in-patient organisations, health care practitioners and policy makers, helping them to come together and drive meaningful change.

## Definition and type of digital health interventions

In 2018, the World Health Organization (WHO) presented a classification of digital health interventions (DHIs) [29]. This taxonomy classifies digital health solutions according to the health system challenges they seek to address. In contrast, the United States Federal Drug Administration (FDA) defined a digital health taxonomy based on the product or service to guide legal regulations for each product [30]. Other existing classifications use the target group (e.g. clients, healthcare providers, health system managers or data services) [31] for the purpose of the intervention [32]. In the context of this roadmap, we used the WHO classification and focused on client or provider-facing technologies and data services.

## Potential of digital health in preventing and managing cardiovascular disease

Several DHIs have shown potential for CVD management [18,33,34,35,36,37,38,39,40,41,42,43,44], including text message programmes [34,38,39,40,41,42,43], mobile (mHealth) apps [18,33,35,36,44], telehealth consultations [45,46,47], wearable devices [48,49,50], and electronic decision support tools [37,51,52]. This section provides several non-exhaustive examples of DHIs to illustrate their potential impact. Text message programmes can improve the management of single risk factors, including tobacco smoking [33], high blood pressure [34], physical activity [38], weight management [39], and medication adherence [40,41]. For example, the TEXTME randomised controlled trial (RCT) showed that four semi-personalised messages sent automatically on random times and days of the week significantly improved blood pressure, body mass index, smoking rates, physical activity and adherence to dietary guidelines [42]. A significant part of its success was the co-design of text content with patients and clinicians, using techniques from behavioural psychology [43].

A 2018 systematic review found that mobile (mhealth) apps can reduce rehospitalisation, improve patient knowledge, quality of life, and psychosocial well-being, and help manage CVD risk factors [44]. Importantly, the success of mHealth interventions depended on simplicity, credible and evidence-based information relying on behaviour change concepts, real-time data tracking, virtual positive reinforcement, app personalisation, social elements, and ensuring privacy [53]. The HERB Digital Hypertension 1 (HERB-DH1) RCT (390 patients from 12 sites in Japan) showed that an interactive smartphone app retrieving home BP monitoring data to generate a personalised programme of lifestyle modifications improved ambulatory, home and office SBP [35].

Physical activity trackers are an example of wearable devices that may be used to support CVD management. A 2019 systematic review (28 RCTs including 3646 participants across nine countries) investigated the effects of wearable activity trackers and found an average increase of 627 daily steps (95% CI 417 to 862 steps) and energy expenditure among the intervention groups compared to controls [50]. The mSToPS RCT demonstrated that a home-based wearable continuous ECG monitoring patch could successfully identify patients with atrial fibrillation [54]. The Apple Heart Study demonstrated moderate effectiveness of the Apple Watch in identifying people with atrial fibrillation [55]. However, this study was not without criticism [56]: An essential issue was the absence of a response by individuals notified by the Apple Watch of possible health issues, highlighting the importance of considering how users interact with DHIs to ensure effectiveness.

The EXPERT (Exercise Prescription in Everyday Practice & Rehabilitation Training) Tool is an example of a clinical electronic decision support tool. The European Association for Preventive Cardiology (EAPC) introduced EXPERT as an interactive decision aid enabling healthcare professionals to compare their exercise prescriptions to predetermined patient cases using an algorithm [57]. The recent PROMPT-HF trial showed that a tailored EHR-based alerting system could improve the use of guideline-directed medication in heart failure [52]. Lastly, artificial intelligence (AI) decision support tools can aid in interpreting medical imaging data automatically [51,58,59].

## Requirements for successful implementation of digital health solutions

The WHO recommendations on DHIs for health system strengthening laid out several conditions necessary to implement digital health technologies successfully [60]. These include (1) The health content, referring to the disease domain and associated treatment recommendations, (2) a DHI that is technically aligned to achieve the proposed goal (3) digital applications such as information and communications technology (ICT) systems and communication channels to deliver the digital intervention — these include the ICT and software systems optimised for its intended goal — and (4) an enabling environment consisting of a national strategy, reimbursement policies, and a digital infrastructure. A recent European Society of Cardiology (ESC) working paper emphasises the importance of DHIs for managing CVDs and provides various practical recommendations [12].

## Roadblocks and solutions to implement digital health interventions

**Figure 2:**
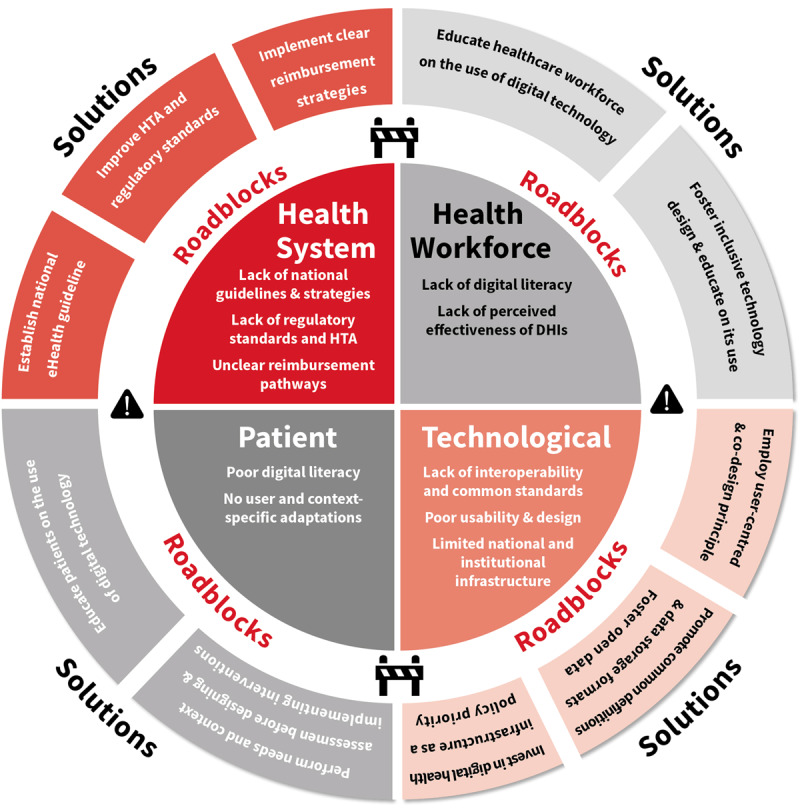
Selected roadblocks and solutions to implement digital health interventions, based on the WHO/ITU framework. *© World Heart Federation*.

Unfortunately, in reality, many of the ideal conditions for implementing digital health technologies are often not met. The WHO/ITU framework suggests that a national eHealth environment requires good leadership and governance, high-quality legislation and policies, a clear investment and reimbursement strategy, high-quality services and applications, a supporting digital health infrastructure, clear interoperability and data standards and a tech-savvy workforce [61]. Barriers and solutions were classified according to these components. In addition, a category of patient-level barriers was added as it was not included in the WHO/ITU framework (Figure 2).

Table 1 outlines common barriers to implementing digital health solutions based on a narrative synthesis of existing literature and the online survey. The online survey was shared with WHF membership organisations and social media channels between September 2, 2021 and October 14, 2021. In total, 227 participants from 71 countries completed the survey. The survey achieved global representation: 28.4% of the responses were collected in the WHO European region; 25.3% in the Americas, 21.3% in South-East Asia, followed by 12% in the African Region, 9.8% in the western Pacific and 3.1% in the Eastern Mediterranean regions. Furthermore, 35% were from high-income countries, 60% from middle-income countries and 15% from low-income countries, according to 2019 World Bank Criteria. Solutions were identified based on expert consensus and existing literature. In the next section, we discuss some of the most reported barriers to implementing digital health solutions and provide solutions that might be relevant to the local context.

**Table 1:** Barriers and possible solutions.


	DESCRIPTION OF ROADBLOCK	SOLUTION

**Leadership and governance**		

National guidelines and strategies	Lack of national guidelines and eHealth strategy.	Establish national or regional eHealth guidelines and strategy.

Stakeholder engagement	Poor involvement of critical national stakeholders.	Inclusive engagement with stakeholders by policymakers, including representatives of patients, practitioners, payers, industry and civil society.

Monitoring and evaluation standards.	Lack of clear monitoring and evaluation standards. No repeated monitoring of effectiveness, reach and impact of interventions.	Clear national standards for monitoring and evaluation of DHIs. Long-term monitoring of effectiveness and implications of digital health interventions. ‘unexpected effects’ registry.

**Legislation, policy and compliance**		

National legislation on data security and access	Lack of national guidelines on data security and access. Local institutional guidelines are not harmonized.	Explicit national guidelines on data access and security. Promote harmonization of policies between institutions.

Lack of regulatory approval or guidance	Lack of regulatory standards; poor health technology assessment (HTA) standards.	Improve HTA and regulatory standards.

**Strategy and investment**		

Reimbursement	Unclear reimbursement pathways for digital technologies.	Clear reimbursement strategy for DHI. Include economic evaluations in the design phase.

Long-term investment strategy	Lack of long-term investment strategy for sustainability of digital technologies.	Include long-term investment strategy as part of national guidelines.

**Services and applications**		

Contextualisation	Intervention not adapted to the local context.	Perform a structured and holistic needs and context assessment before designing and implementing interventions. Health system assessment frameworks might be helpful tools.

Poor usability and design	Non-user focused design.	Employ user-centred and co-design principles. Include end-users (practitioners/patients) early in the design phase.

**Infrastructure**		

National or regional digital infrastructure	No clear investment in national or regional digital infrastructure.	Investing in digital health infrastructure should be included as a national policy priority.

Healthcare provider systems	Local infrastructure does not allow the integration of new DHI.	Applications should be flexible and available in on- and offline modes.

**Standards and interoperability**		

Data structure standards	National and international differences in data collection, storage and definitions standards.	Promote collective definitions and data storage formats. Emphasise implementation of open data platforms.

**Health workforce**		

Poor needs assessment	Poor understanding of the health workforce needs.	Include clear health system and needs assessment in the design phase of DHIs.

Data literacy	Lack of understanding of DHI.	Provider education on the use of digital technology.

Low acceptability	Lack of perceived effectiveness and use of DHIs.	Inclusive technology design and education of use.

**Patients**		

Poor digital literacy and skills	Lack of understanding of DHI (literacy), or not having physical capabilities to interact with DHI.	Patient education on the use of digital technology, context specific adaptations of technology to match patients’ physical abilities.

Low acceptability	Lack of perceived effectiveness and use of DHIs.	Inclusive technology design, education of use and user acceptance, usefulness and engagement evaluation alongside clinical trials and related research.


### Leadership and governance

#### Roadblock: Lack of national guidelines

National governments and policymakers are uniquely responsible for setting universally applicable strategies and standards [61]. More than 70% of respondents to the survey noted that a lack of national data privacy and sharing guidelines was a critical roadblock. The lack of national guidelines was an essential barrier in a recent systematic scoping review [16], as it can lead to the reluctance of providers to use DHIs. For example, the absence of clear national guidelines on telehealth solutions in Bangladesh was a significant barrier for local healthcare practitioners to adopt this technology [62].

##### Suggested solutions

The ITU/WHO national eHealth strategy toolkit guides the development of national eHealth strategies [61]. National professional medical societies are uniquely positioned to advocate for national guidelines on data sharing and universal data standards. Increasingly, digital health data governance standards are emerging, involving regulations for ethics, data security, and regulatory policies [63]. The development and deployment of digital health technologies are dependent on the ability to collect, store, access and share medical data. Clear guidelines for accessing these data can ensure their quality and availability [64]. A key aspect of development includes rigorous stakeholder management with an emphasis on inclusive development [61].

##### Roadblock: Lack of monitoring and evaluation standards

In the WHF digital health roadmap survey, low (perceived) quality was a moderate to significant barrier to more than 70% of respondents. The WHO reported that only 7% of DHIs in LMICs are properly evaluated [65]. A systematic review confirmed these results and found only the minority of DHIs are properly evaluated [66]. Similarly, few AI decision support tools are evaluated prospectively [67]. On a policy level, few countries have dedicated frameworks for evaluating DHIs [51]. The lack of perceived or proven effectiveness is a critical barrier to the use of DHI by patients and clinicians [16]. The lack of proper evaluation standards has prevented the uptake of DHIs in clinical guidelines, which has negatively influenced adoption. Because they imply multiple interacting components that can target various behaviours and require significant expertise to deliver or target numerous groups of people, many DHIs can be considered complex interventions as defined by the British Medical Research Council [68]. This means that the evaluation of DHIs in RCT settings is complex and not always possible when deployed in a ‘real-world’ setting. Software-driven by AI algorithms poses a unique challenge. These algorithms often undergo continuous training to improve their predictive capabilities. However, there is a significant risk that ongoing training reinforces existing structural biases in the data [69].

###### Suggested solutions

The WHO has highlighted the importance of robust evaluation and recommends using the mHealth Evidence Reporting and Assessment (mERA) checklist, emphasising quantitative, qualitative, and economic evaluation [70,71]. The United Kingdom National Institute for Health and Care Excellence (NICE) provides an Evidence Standards Framework for DHIs [72]. Cardiology guidelines have a hierarchy of evidence that puts RCT at the top. Rarely are digital technologies assessed this way – partly because of the small nature of many companies developing tech, and the complex and rapid nature of DHIs. This requires a shift in the mindset of appreciating DHI’s effectiveness. Many DHIs are considered complex interventions with multiple interacting components. For example, the mWellcare Cluster-Randomized Controlled Trial randomised community health centres and compared the use of electronic decision support tools versus care as usual [73]. The nature of the intervention required healthcare practitioners (doctors, nurses) to engage with the software effectively. A process evaluation can evaluate whether the lack of benefit of mWellcare was due to poor staff engagement with the software or to the software’s intrinsic capabilities. Frameworks for evaluating complex interventions, such as the RE-AIM framework [74] can be used to assess complex DHIs with multiple interacting components. Evaluation of AI-drive software requires specific frameworks [75]. The United States Food and Drug Administration (FDA) outlined a framework in their AI action plan [76]. This framework requires a predetermined change control plan with anticipated algorithm changes by the developers and how this would impact safety and performance [76]. Subsequent post-market access periodic updates are necessary to monitor real-world performance [76]. Professional medical associations play a role in advocating for the necessity for continuous monitoring of AI-drive algorithms. In the same way that post-market monitoring of drug safety and effectiveness is performed in phase IV trials, we require continuous monitoring of software performance and safety when using rapidly changing algorithms.

### Legislation, policy and compliance

#### Roadblock: Lack of guidance on data security of digital health technologies

Privacy concerns and individuals’ willingness to disclose personal medical information are essential barriers to adopting technologies [77]. The lack of national regulations and inadequacy of legal requirements of DHIs was considered a significant barrier by more than 60% of respondents of the WHF survey. Multiple studies have shown that this is particularly true for older individuals [78] and is essential in creating a ‘digital divide’. A study in Ireland suggested that perceived trust and safety of digital (mHealth) technologies were important adoption determinants, particularly in older individuals [79]. Similar studies performed in India and China indicate that trust in the data security of digital health technologies contributes to digital health adoption by patients across cultures and geographies [80,81]. A recent systematic review highlighted the importance of the lack of clarity of digital health regulations as a significant barrier in LMICs [82]. Results of the online survey support these findings: lack of national rules and issues surrounding data privacy for clients (patients) were among the most significant barriers to implementing digital technologies. A recent scoping review highlighted a lack of trust and issues with data privacy and security as a vital clinician-level barrier in 7% of studies on barriers and enablers for digital health in CVD [16]. A recent study from the United Kingdom suggested that a National Health Service (NHS) stamp of approval served as a significant facilitator for digital health implementation [83].

##### Suggested solutions

Rigorous and transparent regulatory mechanisms and guidance on data security and privacy are essential conduits to enhance trust by patients and clinicians in using digital health technologies [61,64]. An article by Tiffin and colleagues summarises components of data governance. It includes guidelines on ethics and consent, data access, sustainability, and legal frameworks on data security, third party access and a right to privacy [84]. The European Union’s General Data Protection Regulation (GDPR) outlines vital data protection principles as a global gold standard [85]. Importantly, patients are increasingly asking to be the owners of their data. In this situation, the patient determines with whom their data is shared and in what context, even after informed consent. While this is not commonplace, new technologies such as blockchain might facilitate individual patient data ownership for research and development purposes [86].

### Strategy and investment

#### Roadblock: No strategy for reimbursement and long-term investment

Current policy guidelines in many countries require face-to-face consultation for reimbursement. Therefore, the lack of proper reimbursement of DHIs is a significant barrier to adoption by patients and providers. More than half of survey respondents considered limited reimbursement, prohibitive costs of training staff and patients, and high out-of-pocket patient costs significant barriers. Separate scoping and systematic reviews [16,87,88] on barriers and facilitators highlighted that financial concerns were essential barriers for both patients and health care providers. Unfortunately, many countries’ care facilities or suppliers’ costs are not reimbursed [12]. Furthermore, the financing of DHIs often does not consider long-term sustainability. In Africa, 85% of the funding for DHIs is targeted at research and early pilot programmes [89,90]. For example, a national survey in Uganda found that most DHIs were pilot studies, operated in silos, donor-funded, and lacked sustainability [91]. Unfortunately, donor-based funding in LMICs often does not consider financial sustainability [92], and long-term public financing by public healthcare systems in LMICs is often challenging [89].

##### Suggested solutions

Implementation of DHIs should consider funding mechanisms because they will impact the (cost) evaluation and long-term sustainability. The COVID-19 pandemic has accelerated the need for new funding mechanisms for telehealth solutions, for example, in the United States [93] and Singapore [94]. Unfortunately, these funding mechanisms have primarily been ad-hoc. Some of these funding mechanisms have been made permanent, as in Australia for example [95]. Governments play a crucial role as conveners to guide the long-term financing strategy, recognised by the WHO/ITU framework for developing a national eHealth strategy [61]. National financing strategies should consider the national and local IT infrastructure as fundamental perquisites for implementing individual DHIs. While many governments have funded telehealth services with health professionals and electronic pharmacy scripts, this does not extend to using DHIs such as apps and wearable devices [51]. Specific financing instruments are beyond the scope of this roadmap. Examples relevant to the local context can be found in the Broadband Commission for Sustainable Development Working Group on Digital Health report [89]. Therefore, researchers/developers should consider the long-term financial sustainability of DHIs during the design phase and cost-effectiveness analyses during the testing phase [68].

### Services and applications

#### Roadblock: No user- and context-specific adaptations

Common issues with DHIs are insufficient assessment of the patients’ and healthcare providers’ actual needs, developing ‘one-off’ interventions without contextualisation within national health systems, and lacking cultural and social adaptations [96]. Lack of contextual adaptation was considered a barrier to DHIs among more than 60% of survey respondents for this roadmap. The effectiveness of DHIs is dependent on the local context, which can also include the wider socioeconomic context. For example, a recent evaluation of a mobile text-based support programme for people living with diabetes or hypertension in Cambodia found that the intervention added little to an already effective peer-support network. Notably, the intervention did not address the structural barriers determining access to care, such as patient reimbursement and patient health literacy [20]. DHIs should also be designed with the user in mind. The introduction of electronic medical records designed without considering the clinician’s workflow is one of the prime examples of poor design standards [97]. Increased work and responsibilities for clinicians were the most significant barrier to the uptake of digital health technology by cardiovascular clinicians in a recent scoping review [16]. Similar to patients, poor design and ability to interact with DHIs were significant barriers to adoption among healthcare workers [16,98].

##### Suggested Solutions

There is a need to focus on unmet population needs and promote thoroughly researched and adequately contextualised technologies. Contextualising DHIs starts by recognising that many are often considered complex interventions [68]. DHIs involve various components that interact within the health system and broader sociopolitical context. For example, mobile app interventions in rural areas with poor reception and internet connectivity will likely not enable community health workers to provide better care. Therefore, contextualisation requires a system thinking approach to recognise the complex interacting parts in determining access to and quality of care in the local context [99]. This should translate to a broad needs and contextual assessment, considering the local (ICT) context, workflow, and sociopolitical barriers to accessing high-quality care. Health system assessment tools and frameworks can help structure the review of the current obstacles and facilitators surrounding CVD prevention and care [100,101], which often require a mixed-methods approach. User-centred and co-design principles are necessary to contextualise interventions and make them more effective [102,103]. The World Wide Web Consortium summarised the user-centred design process [104], which outlines clear steps in the (co)-design of applications. The International Organization for Standardization (ISO) provides further guidance on human-centred design for interactive systems [105].

### Infrastructure

#### Roadblock: Limited national and institutional ICT infrastructure

Limited national and institutional ICT and digital infrastructure are essential barriers to implementing DHIs [61,106]. In 2020, almost 40% of the global population did not have access to the internet [107]. Lack of internet access and institutional support were moderately or very important barriers according to more than 50% of survey respondents in determining access to DHIs. Poor internet connection remains a significant barrier in many LMICs [16,108]. Institutional support and existing infrastructure were critical enablers [16]. ICT infrastructure is also a key barrier on an institutional level. Many LMICs still have paper-based records and limited existing ICT infrastructure [109,110]. The 2016 WHO atlas on eHealth Country Profiles demonstrated that only 10% of 125 countries used EHRs [110]. A national assessment of barriers to implementing digital technology interventions to improve hypertension management in India found that IT infrastructure was available in less than half of mid-tier primary and community health centres [111].

##### Suggested Solutions

DHIs require sufficient and efficient information and communications infrastructure, including software, hardware, internet connectivity, maintenance support, data storage and security. National governments should consider national infrastructure in their eHealth and finance strategy, for which the ITU/WHO guidance is a valuable tool [61]. In addition, the WHO and ITU have developed a ‘digital Health Platform Implementation Handbook’, which can serve as a guide to implementing digital health platforms [112]. On an institutional level, national society guidelines might help guide implementation. The American Medical Association developed a playbook for implementing telehealth, which provides a practical guideline for healthcare systems to implement telehealth consultations [113]. Almost all the public system innovations meant to be adopted require long-term support and maintenance planning to ensure the product’s sustainability after the initial implementation phase. Researchers/innovators working on digital health solutions should try to align their products with national policies and infrastructure. During the design phase of a DHI, researchers and producers should consider local infrastructure requirements. This requires flexibility of the application. In many LMICs, applications should be able to function as stand-alone services and in offline settings.

### Standards and interoperability

#### Roadblock: Limited interoperability of digital health interventions

Lack of integration with the existing (ICT) workflow was a moderate to significant barrier for most respondents. These results were in line with published studies and meta-analyses [91,92]. Institutional data is often saved digitally or in paper format and, depending on the institution, usually uses proprietary software solutions. Cardiac imaging analyses are stored in Digital Imaging and Communications in Medicine (DICOM) format in some institutions but as OpenCV or MP4s in others. Lack of technical interoperability can prevent data exchange between two technologies, such as electronic medical records and a new AI application. The Global System for Mobile Communications Association (GSMA) emphasised that legacy ICT infrastructure and commercial software packages using non-open data standards commonly cause a lack of interoperability [89]. Enhancing interoperability is critical to enabling training and deployment of algorithms and a substantial barrier to scaling DHI nationally [92]. The lack of high-quality data in LMICs can perpetuate healthcare disparities [114]. Without access to high-quality large volumes of data, it is challenging to develop effective algorithms for populations in LMICs.

##### Suggested Solutions

The Health Data Collaborative Digital Health & Interoperability working group, currently co-chaired by USAID and WHO, has developed several toolkits relevant to developing and implementing national data interoperability standards [115]. Many data platforms are available, which can also function as (national) medical record platforms. Examples include OpenDataKit [116], OpenMRS [117], and CommCare [118]. In 2014, a randomised study in South Africa demonstrated that CommCare had no errors compared to 3.8% errors in the paper-based arm for assessing CVD risk [119]. The broadband commission working group on digital health highlighted that LMICs without legacy systems are uniquely positioned to leapfrog high-income countries by adopting new solutions faster [89]. Professional societies can help promote the importance of interoperability standards. For example, the European Society of Cardiology (ESC) launched the Cardiology Audit and Registration Data Standard in 2015 to promote the collection of shared data definitions in health information systems [120].

### Health workforce

#### Roadblock: Acceptability and feasibility for health workers

DHIs have the potential to empower and educate health workers, reduce their workload and save travelling time [60]. A recent scoping review identified the most common barriers health workers face in CVD [16]. These included increased workload and responsibilities, commonly due to poor integration with existing ICT systems, unreliable technologies and/or lack of evidence supporting using the technology, financial concerns with using the technology, and data privacy and security concerns. Similar reviews studying barriers and facilitators for DHIs for hypertension management highlighted identical obstacles, including lack of technology usability and support, lack of validation of technology and concerns over data privacy and security [87,88]. One of the most significant barriers to implementing DHIs is interventions not working within existing clinical workflows and poor usability. Reimbursement of digital health technologies impacts adoption by healthcare workers. A recent McKinsey survey highlighted that many patients embraced telehealth visits, but physicians still had significant reservations [121]. Particularly, a minority of physicians felt that telehealth visits were more convenient than most patients [121]. Finally, healthcare workers’ digital literacy and confidence in using newer digital health technologies might be a significant barrier. A recent survey on e-health knowledge and usage in general cardiology by the European Society of Cardiology (ESC) Practice and Digital Health Committee suggested that more than 25% of cardiologists rated their knowledge of eHealth as low [122].

##### Suggested solutions

User-focused design and a solid evidence base regarding effectiveness are vital in enabling clinicians to use digital health technologies [60]. As a rule, digital technologies should reduce the burden on clinicians or significantly improve patient outcomes to be acceptable. Involving healthcare workers in the design phase of DHIs can prevent poor usability and integration with existing clinical workflows. Most DHIs are not evaluated in randomised controlled trials [66]. Therefore, the design of DHIs should be accompanied by rigorous testing in randomised clinical trials. Digital literacy of health workers is vital. Therefore, targeted health worker education on using digital health technologies is essential. Notably, costs of (continued) training of healthcare workers in digital technologies should be considered when estimating cost-effectiveness. Professional medical organisations, such as ESC, AHA or the Asia Pacific Society of Cardiology, have a unique opportunity to educate their members on the benefits and limitations of DHIs and their evaluation. Specifically, they could offer training during professional meetings on digital solutions and develop specific eLearnings for their members.

### Patients

#### Roadblock: Acceptability and feasibility for patients

Similar to providers, DHIs have the potential to empower and educate patients, reduce their workload and save travelling time. Various patient-level barriers impact DHI adoption and acceptability. Patients with CVD are commonly older and have several comorbidities leading to impaired physical and mental functioning [82]. The disease progression of various subtypes of CVD, such as heart failure, can lead to progressive cognitive impairment, which can reduce patients’ ability to engage with DHIs. This barrier is shared across countries of different economic levels [16,82,88,91]. Cultural, and socioeconomic factors might influence the acceptability of digital health technologies. Well-known user characteristics associated with lower digital health use are sex [62], older age, low (health) literacy [62] and low socioeconomic status [123,124,125]. These characteristics, in part, are related to lower digital literacy, especially in the use of mobile apps and telehealth solutions [123,124,125]. For example, patients might feel uncomfortable with home visits or telemedicine visits with video calls in some cultures due to privacy concerns regarding their home situation. Economic barriers remain a significant challenge for patients but have been extensively discussed in previous sections. Finally, a lack of perceived effectiveness experienced by patients and a potential overload of DHIs might hinder adoption. Because patients with CVD often have multiple comorbidities, there is an inherent risk of being confronted with different unrelated DHIs for individual comorbidities (e.g., hyperlipidaemia, hypertension). Patients with a lower education level are at a higher risk of not being able to engage with digital health technologies, especially at an older age [126].

##### Suggested solutions

As is the case for health care workers, digital health technologies require significant investments in patient education. Active involvement of caregivers and assisting patients in using DHIs might improve utilisation [82]. Deployment of DHIs requires a rigorous context and needs assessment. Patient factors such as socioeconomic status, gender role influencing access to technology, cultural factors, practical ability, and digital literacy should be considered. Involving patients in co-designing DHIs is necessary to mitigate these risks and has been demonstrated to be impactful [43]. Further, it is essential to evaluate user perspectives and engagement alongside clinical trials to fully understand how to optimise usefulness and engagement beyond the trial [36].

## Conclusions and recommendations

DHIs can fill critical health system gaps in CVD management and prevention, empower patients, and enable healthcare practitioners to provide higher quality and more efficient care. There is a substantial unmet need for collective action involving patients, healthcare providers, industry members, regulators and reimbursement authorities, and policy makers to identify and help solve context-specific barriers. This roadmap provided potential solutions for frequently encountered obstacles to scaling up digital health solutions to improve the management and prevention of CVD and the support and experience of care for those living with those conditions globally.

## Case studies

### mPower Health a Clinical Decision Support System for Evidence-based Care

#### Health system challenge

Empower non-physician healthcare workers through the use of a digital platform (evidence-based Clinical Decision Support System).

#### Intervention

**mPower Health** is a digital platform designed to deliver integrated, comprehensive, and continuous care to patients through innovative technology-driven tools that support healthcare professionals.

In 2010, the Government of India initiated the National Programme for Prevention and Control of Cancer, Diabetes, Cardiovascular Diseases and Stroke (NPCDCS) to respond to the challenges related to the high burden of NCDs in India. The mPower Health platform was designed to complement the NPCDCS programme.

**Figure 3:**
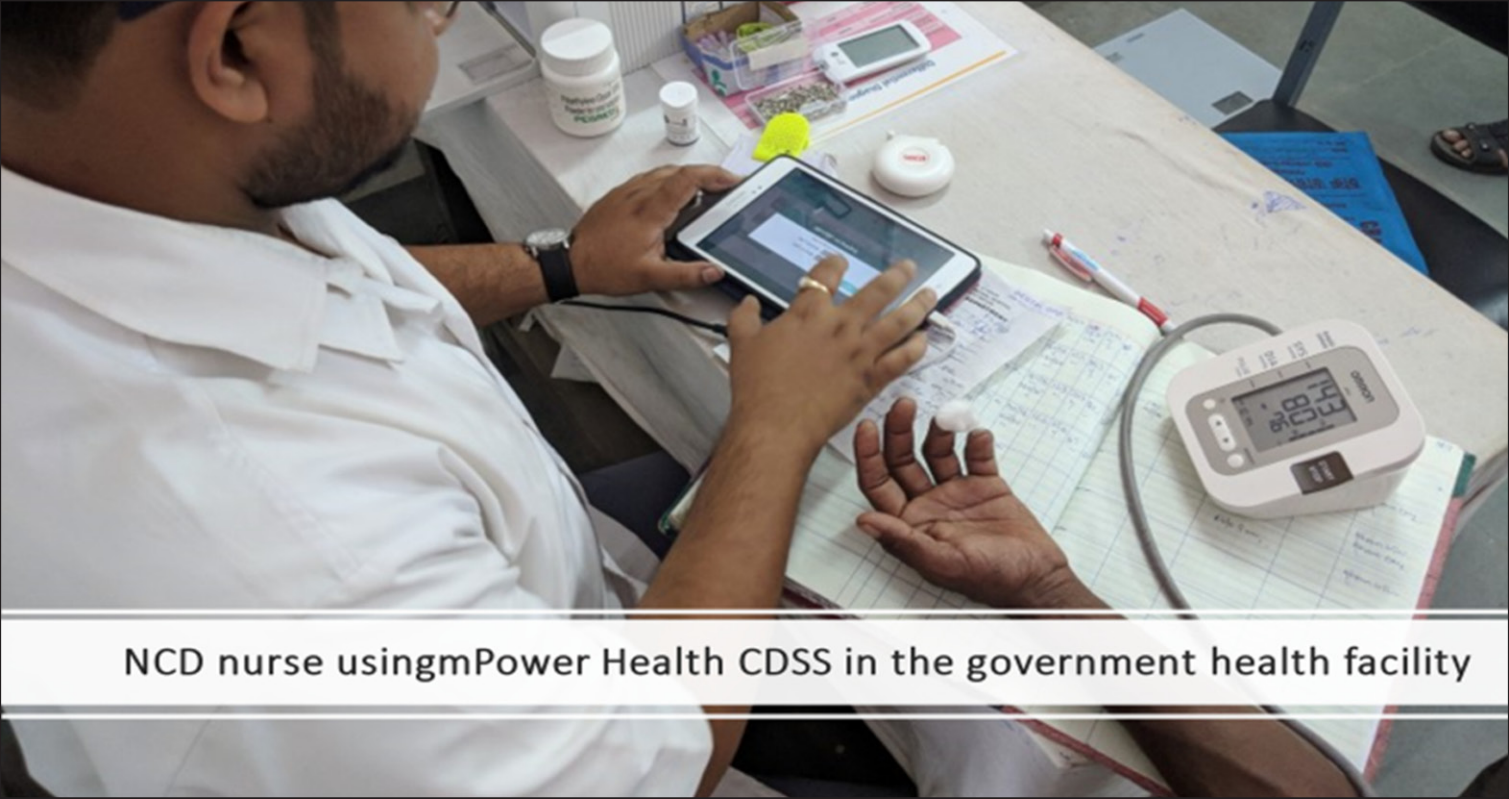
NCD nurse using mPower Health CDSS in a government health facility, reproduced with permission from the Centre for Chronic Disease Control (CCDC), New Delhi, India.

The mPower Heart works on two core principles: Technology (a knowledge-based Clinical Decision Support System-CDSS) and task-shifting (empowering non-physician healthcare providers) (Figure 3).

mPower Health’s major capabilities/features are:

- A mobile app for healthcare providers and a web-based dashboard/server for healthcare administration.
- Clinical Decision Support System generates ersonalized management plans for patients with hypertension and diabetes by computing complex clinical management algorithms and suggesting the diagnosis, optimal drugs, dosage, warning contra-indications, etc.
- Computing clinical risk scores to identify high-risk individuals and initiate preventive measures.
- Maintaining longitudinal health records of the patient and assisting in scheduling follow-up visits based on clinical parameters (avoid unnecessary travel and visits of the patients).
- Task Shifting: empowering the non-physician workforce to deliver quality care by using technology.
- Generating lifestyle recommendations tailored to individual patients.
- Ability to work in offline mode (without internet connectivity).
- Real-time monitoring and profile visualiser for trending and quick decision making.

#### Link with barriers and solutions

##### Acceptability and feasibility for health workers, ICT infrastructure

Piloting of the mPower platform has shown impressive results and significantly impacted clinical outcomes of patients with hypertension and diabetes [127]. Two states (Tripura and Mizoram) governments in India adopted the mPower Health system for state-wide implementation. It was successfully implemented in forty health facilities in the state of Tripura [128] and sixteen health facilities in Mizoram in India with the support of key stakeholders by providing an enabling environment (healthcare workforce, digital infrastructure, change management, etc.). The mPower Health met all the important conditions recommended by WHO for a successful DHI such as health content (mPower Health provided standardised evidence-based care for NCDs), alignment of the intervention with the national goal, digital infrastructure (support provided the state health department) and enabling environment. In Tripura and Mizoram, around 207, 000 people have benefited from this technology-enabled NCD care.

##### National guidelines and clear strategy for long-term investment

The lack of national guidelines and strategies for the DHI at the time of implementation of the mPower Health and no strategies for long-term investment impacted the uptake and sustainability of mPower Health. As a result, the Government of India recently initiated the Ayushman Bharat Digital Mission (ABDM) which aims to provide the necessary framework and infrastructure [129]. The learnings from the states of Tripura and Mizoram and the support provided by national agencies led to integrating the CDSS module of the mPower Health with the Government of India’s Comprehensive Primary Health Care -NCD system under the I-TREC [130]. Furthermore, the World Health Organization (WHO) adopted the mPower platform to develop the mPEN App for promoting the use of the WHO PEN Package in the clinical management of non-communicable diseases.

### CONNECT: a consumer-focused, responsive and primary care-integrated web-application

#### Health system challenge

To integrate data and communication directly between patients and their health care providers.

#### Intervention

**Figure 4:**
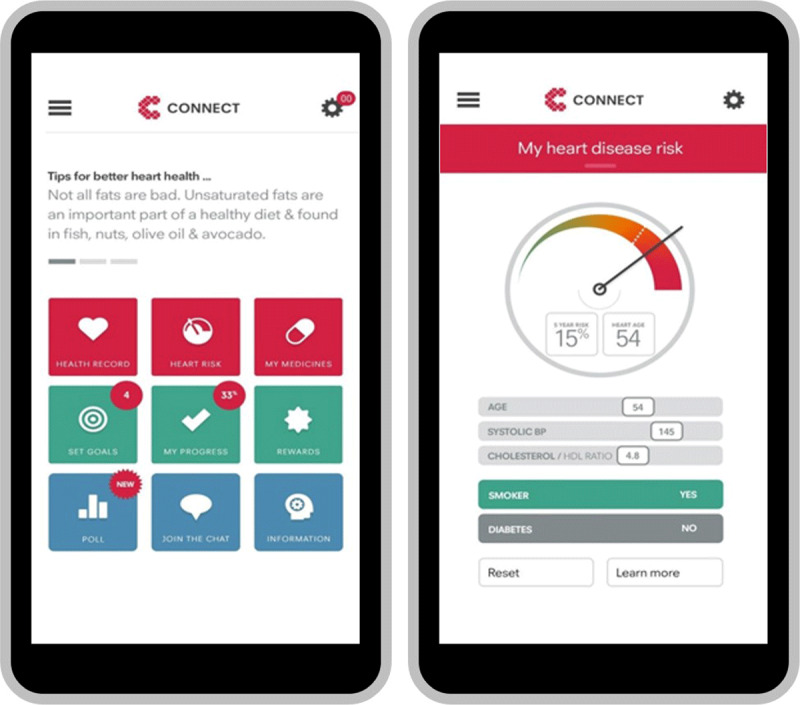
**CONNECT smartphone application.** Reproduced from NPJ Digit Med. 2020; 3. Redfern J, Coorey G, Mulley J, et al., A digital health intervention for cardiovascular disease management in primary care (CONNECT) randomized controlled trial.

Consumer Navigation of Electronic Cardiovascular Tools (CONNECT) is a consumer-focused, responsive web application that is interactive and integrated with data from the patient’s primary care electronic health record. It was co-designed with consumers, clinicians and software developers and supported CVD risk management and decision-making. CONNECT (Figure 4) includes digital reminders and access to (1) medical conditions, medicines and interactive absolute risk awareness (red tiles); (2) goal-setting, progress tracking and virtual rewards (green tiles); polling for interactivity and social interaction (blue tiles). In the words of a patient, *‘It was a nice way of seeing the graph of the cholesterol thing coming down, so that was great–that’s a bonus…visual feedback…I better keep walking, riding, whatever because it’s working.’* [131].

#### Links with barriers and solutions

##### User- and context-specific adaptations

Patients have electronic access to auto-populated information about their medical conditions and prescribed medicines with links to more detailed information to enhance knowledge. CONNECT also has smartphone and website access to an interactive and personalised CVD risk system where individual patients can use interactive functions and receive visual feedback about the relationships between their risk factors and absolute cardiovascular risk. Interactive goal-setting (including virtual rewards) based on healthier eating, physical activity, smoking cessation and emotional well-being, and goal achievement tracking with virtual rewards to facilitate and motivate lifestyle changes.

##### Interoperability of digital health interventions

CONNECT is integrated with each patient’s primary care electronic record. Progress tracking combines data imported from electronic medical records with patient logs such as blood pressure control and weight management with calendar links for tests such as cholesterol measurement requirements.

##### Acceptability and feasibility for healthcare works and patients

The intervention was co-designed, validated and beta-tested via a four-phase iterative process that involved consumers, multidisciplinary clinicians, software developers and graphic designers [132]. Through this process, researchers understood the complexity of end-user needs and preferences, thereby improving and enriching the increasingly detailed system designs and prototypes for a mobile responsive web application. The CONNECT intervention was subsequently tested in a randomised clinical trial (n = 934 patients with or at high risk of CVD) in the Australian primary care environment with an average follow-up of 12. The study found no significant difference between groups for medication adherence, modest (but not significant) improvements in risk but significant improvements in attainment of physical activity targets and e-health literacy [18]. However, in qualitative analysis, patients, reported benefiting from the cardiovascular risk score, goal tracking, risk factor self-monitoring and receipt of motivational health tips. In contrast, general practitioners reported increased patient attendance and engagement with care [131]. CONNECT has also been found to be accessible, well utilised, and supported [121,123]. It includes social interaction and an optional messaging service (email or text). Patients can receive semi-personalised cardiovascular disease prevention tips and motivational messages related to diet, medications and lifestyle. Further work is underway to be iterative in implementation and revision of CONNECT and related DHIs that enable delivery of tools that are useful for patients and beneficial for health outcomes.

### Rural emergency telemedicine support

#### Health system challenge

Deliver emergency care in underserved areas and remote settings.

#### Intervention

Providing emergency care in rural areas continues to be a global challenge. The pandemic amplified the disparity in the care of underserved populations in the United States, notably the 20% of the people living in rural areas. With the closure of many rural hospitals, the emergency medical response system faced longer response and transport times, delaying hospital-level medical care. The Emory rural Tele-Emergency Medical Services (Tele-EMS) network underwent a significant innovative change with the work of Drs. Monique Smith and Michael Carr and their team bring the clinical understanding of the hospital to the point of emergency contact and automate data communication, allowing earlier patient-focused clinical care in the community [133]. The digital strategy needed to accomplish this network in a rural area was an impressive and globally relevant challenge to overcome. It involved three key pillars: clinical expertise, the coordinated transmission of medical information and investment in communication infrastructure.

In this programme, an emergency physician provides a video televisit to rural Grady Hospital EMS crews to evaluate and suggest management of initial patient care. Patient data is uploaded to a streaming cloud if specialised care is needed. The receiving facility is notified of the patient’s arrival and the treatment plans that have been started. This allows emergency medical personnel to focus their attention on the patient’s care while the communication is automated.

#### Links with barriers and solutions

##### User- and context-specific adaptations, ICT infrastructure

In contrast to usual telemedicine programmes, rural platforms require the ability to work in low bandwidth settings and inside and outside the hospital. After evaluating solutions from multiple companies, Emory chose SWYMED [134], which allows telemedicine visits to be conducted with transmission as low as 60 kb per second. This significantly increased the geographic reach of telemedicine utilisation for emergency services. In addition, Emory worked with the primary cellular provider for their region, Verizon, to identify the Airlink MG 90 router (Sierra wireless[R] Vancouver Canada [135]) and installed it in their hospital system and affiliated ambulances. This allowed rapid downlink and uplink speeds, Wi-Fi and ethernet access.

#### Interoperability of digital health interventions

Notably, the platform can integrate into EMS and hospital technology to enable rapid cardiovascular data transmission. In this instance, the product integrated explicitly with Zoll, the X series monitor defibrillator used on Grady ambulances and the Emory electronic medical record.

In conclusion, this case underscores the complex human capital, data standardisation, integration, transmission and communications infrastructure needed to achieve scalable and sustainable rural telemedicine. It also highlights the importance of multi-industry collaboration to create meaningful change in rural health by creating new systems for high-quality access to high-quality care.
